# High anal swab viral load predisposes adverse clinical outcomes in severe COVID-19 patients

**DOI:** 10.1080/22221751.2020.1858700

**Published:** 2020-12-24

**Authors:** Haibo Li, Lili Ren, Lulu Zhang, Yeming Wang, Li Guo, Conghui Wang, Yan Xiao, Ying Wang, Jian Rao, Xinming Wang, Ying Liu, Chaolin Huang, Xiaoying Gu, Guohui Fan, Hui Li, Binghuai Lu, Bin Cao, Jianwei Wang

**Affiliations:** a Department of Pulmonary and Critical Care Medicine, Center of Respiratory Medicine, National Clinical Research Center for Respiratory Diseases, China–Japan Friendship Hospital, Beijing, People’s Republic of China; b Institute of Respiratory Medicine, Chinese Academy of Medical Science, Beijing, People’s Republic of China; c NHC Key Laboratory of Systems Biology of Pathogens and Christophe Mérieux Laboratory, Institute of Pathogen Biology, Chinese Academy of Medical Sciences & Peking Union Medical College, Beijing, People’s Republic of China; d Key Laboratory of Respiratory Disease Pathogenomics, Chinese Academy of Medical Sciences and Peking Union Medical College, Beijing, People’s Republic of China; e Department of Respiratory Medicine, Capital Medical University, Beijing, People’s Republic of China; f Department of Tuberculosis and Respiratory Disease, Jinyintan Hospital, Wuhan, People’s Republic of China; g Institute of Clinical Medical Sciences, China–Japan Friendship Hospital, Beijing, People’s Republic of China; h Tsinghua University–Peking University Joint Center for Life Sciences, Beijing, People’s Republic of China

**Keywords:** SARS-CoV-2, COVID-19, viral load, anal swabs, clinical outcome

## Abstract

To identify the association between the kinetics of viral load and clinical outcome in severe coronavirus disease 2019 (COVID-19) patients, a retrospective study was performed by involved 188 hospitalized severe COVID-19 patients in the LOTUS China trial. Among the collected 578 paired throat swab (TS) and anal swab (AS) samples, viral RNA was detected in 193 (33.4%) TS and 121 (20.9%) AS. A higher viral RNA load was found in TS than that of AS, with means of 1.0 × 10^6^ and 2.3 × 10^5^ copies/ml, respectively. In non-survivors, the viral RNA in AS was detected earlier than that in survivors (median of 14 days vs 19 days, *P* = 0.007). The positivity and viral load in AS were higher in non-survivors than that of survivors at week 2 post symptom onset (*P* = 0.006). A high initial viral load in AS was associated with death (OR 1.368, 95% CI 1.076–1.741, *P* = 0.011), admission to the intensive care unit (OR 1.237, 95% CI 1.001–1.528, *P* = 0.049) and need for invasive mechanical ventilation (OR 1.340, 95% CI 1.076–1.669, *P* = 0.009). Our findings indicated viral replication in extrapulmonary sites should be monitored intensively during antiviral therapy.

## Introduction

Coronavirus disease 2019 (COVID-19) is caused by severe acute respiratory syndrome coronavirus-2 (SARS-CoV-2), can lead to severe or critical disease, and is a global pandemic [1,2]. The clinical manifestations of COVID-19 range from asymptomatic infection, to mild, severe or critical respiratory tract infections, gastrointestinal and neurological symptoms, and death [3–7]. SARS-CoV-2 infection is estimated to be responsible for approximately 20% of severe cases and approximately 5% of fatal cases among infected individuals [2]. For this deadly infection, much attention should be paid to decreasing mortality in severe cases with effective antiviral therapies. It is therefore of great significance to precisely determine the kinetics of virus shedding and the sites of viral replication.

The presence of viral RNA has been reported in a broad range of sample types, including but not limited to respiratory, stool, urine, and blood samples [3,7]. Viral RNA detection not only provides major evidence for clinical diagnosis but also reflects the virus replication sites, and the viral RNA load is a useful parameter to identify the status of viral replication and clearance [7–9]. Hence, viral load quantification in patients has been used to monitor disease progression. The correlations between viral RNA load and clinical symptoms and laboratory test results have provided clues to predict disease severity. For example, the viral RNA levels in nasopharyngeal aspirates and blood were correlated with death in SARS-CoV-infected patients [10]. In COVID-19 patients, the viral loads in sputum and blood were found to be related to prognosis [7,11]. However, most recent studies involved few severe cases and employed a single sample type. Whether the viral load in samples collected from different anatomical sites will predict clinical outcome in severe patients still needs to be thoroughly investigated.

In a previous report, two COVID-19 cohorts suffering from severe infections were recruited for a clinical trial (LOTUS) to determine the antiviral efficacy of lopinavir-ritonavir [8]. In this study, we longitudinally quantified the viral load in consecutive throat swab (TS) and anal swab (AS) samples collected from the LOTUS cohorts to evaluate the viral loads in specimens collected from different anatomical sites and their association with clinical outcomes in severe COVID-19 patients. Our findings suggest that viral replication in extrapulmonary sites and viral RNA load are highly correlated with adverse outcome of COVID-19 patients.

## Methods

### Patients and clinical samples

The recruitment criteria for COVID-19 patients and the sampling strategies have been reported previously, and the involved patients were enrolled at Wuhan Jinyintan Hospital, Wuhan, China, from 18 January 2020, through 3 February 2020 [8]. All patients were hospitalized with COVID-19 of grade 3 or more on the seven-category ordinal scale (reported previously) [8].

Among the 199 recruited patients, eight recovered patients and three patients who died were excluded because the relevant clinical samples were not enough to be used for virus detection in this study. The eligible 188 patients were included for further analysis. Of the 188 recruited patients, 147 (78.2%) recovered, and 41 (21.8%) died. A total of 31 (16.5%) patients were admitted to the intensive care unit (ICU), and 27 (14.4%) received invasive mechanical ventilation. The age range was 15–85 years, and the median age was 57.5 years (interquartile range [IQR], 48.8–67.3 years). Male patients accounted for 59.6% of the population (112) (Table 1). The clinical records were collected retrospectively. At the time of recruitment, 26 patients had grade 3, 134 had grade 4, and 28 had grade 5 disease according to the seven-category ordinal scale.

**Table 1.** The demographic information and clinical symptoms of recruited COVID-19 patients.

	Total (cases = 188)	Survivors (cases = 147)	Non-survivors (cases = 41)	*P* value
Age, years	** **	** **	** **	** **
Median (IQR)*	57.5 (48.8-67.3)	56.0 (46.0-65.0)	65.0 (57.0-73.5)	<0.001
Sex, *n* (%)				0.198
Male	112 (59.6)	84 (57.1)	28 (68.3)	..
Female	76 (40.4)	63 (42.9)	13 (31.7)	..
Underlying disease, *n* (%)				
Yes	111 (59.0)	88 (59.9)	23 (56.1)	0.665
Hypertension	65 (34.6)	53 (36.1)	12 (29.3)	0.419
Diabetes	23 (12.2)	20 (13.6)	3 (7.3)	0.277
Heart disease	15 (8.0)	11 (7.5)	4 (9.8)	0.744
Cerebrovascular disease	13 (6.9)	10 (6.8)	3 (7.3)	1.000
Chronic kidney disease	6 (3.2)	5 (3.4)	1 (2.4)	1.000
Malignancy	6 (3.2)	4 (2.7)	2 (4.9)	0.613
Chronic liver disease	8 (4.3)	6 (4.1)	3 (7.3)	0.412
Symptoms, *n* (%)				
Fever	175 (93.1)	139 (94.6)	36 (87.8)	0.161
Cough	160 (85.1)	127 (86.4)	33 (80.5)	0.348
Fatigue	49 (26.1)	40 (27.2)	9 (22.0)	0.498
Headache	7 (3.7)	6 (4.1)	1 (2.4)	1.000
Muscle pain	22 (11.7)	19 (12.9)	3 (7.3)	0.418
Pharyngalgia	6 (3.2)	6 (4.1)	0 (0.0)	0.342
Dyspnea	23 (12.2)	18 (12.2)	5 (12.2)	0.993
Diarrohea	5 (2.7)	5 (3.4)	0 (0.0)	0.587
Severity on the seven-category ordinal scale, *n* (%)				
3	26 (13.8)	21 (14.3)	5 (12.2)	0.732
4	134 (71.3)	106 (72.1)	28 (68.3)	0.633
5	28 (14.9)	20 (13.6)	8 (19.5)	0.348
Antibiotic use, *n* (%)				0.613
Yes	182 (96.8)	143 (97.3)	39 (95.1)	..
Corticosteroid use, *n* (%)				0.294
Yes	65 (34.6)	48 (32.7)	17 (41.5)	..

* IQR, interquartile range. *P* values were calculated by two-sided unpaired *t*-test or *χ*^2^ test as appropriate.

Paired consecutive TS and AS samples maintained in viral transport medium (VTM) were collected on days 1, 5, 10, 14, 21, and 28 after recruitment (until hospital discharge or death). Consecutive TS and AS samples were obtained 5 times in 10 patients, 4 times in 65 patients, 3 times from 55 patients, and 2 times from 45 patients. Only one sample pair was obtained from thirteen patients. A total of 1156 samples (578 TS and AS pairs) were collected for further analysis.

### Procedures

Samples (400 μl) from TS and AS were added into 2 ml lysis buffer in a biosafety level 3 laboratory, and nucleic acids were extracted by using a NucliSENS easyMAG system (bioMerieux, Marcy l’Etoile, France) according to the manufacturer’s instructions. A 50 µl elution was obtained from each sample. The presence of viral RNA and the viral load in the samples were determined by quantitative RT–PCR using a Bio-Rad instrument (Bio-Rad CFX96, Hercules, CA, USA) [13]. As for the sensitivity of open reading frame 1b (ORF1b) genes was lower than that of the nucleocapsid (N) gene, the primers were designed to target the N gene of SARS-CoV-2 [13]. The primer sequences were as follows: F 5’-ACCTGTGTAGGTCAACCACG-3’, R 5’ -CAGCGCTTCAGCGTTCTTCGGAATGTCGC-3’. Nucleic acids (5 µl) were used for RT–PCR, and the conditions were as follows: 15 min at 50°C for reverse transcription, 4 min at 95°C for predenaturation, and then 45 cycles of 15 s at 95°C and 45 s at 60°C. The quantified RNA transcripts for N gene was prepared by *in vitro* transcribed plasmids with a T7 promoter (pEasy-T1, TransGen Biotech, Beijing, China) via *in vitro* transcription with the RiboMAX™ Large Scale RNA Production System (Promega, Madison, WI, USA). The concentration of the RNA transcripts was determined using NanoDrop technology (Thermo Fisher Scientific, Waltham, MA, USA). The subgenomic RNA was tested according to previous report [14].

### Statistical analysis

The data presented as durations were calculated from the onset of symptoms. The consecutive data, including viral load in different specimens and the time for the virus test to turn from positive to negative, were compared by Student’s t-test or the Mann–Whitney U test as appropriate. The categorical variables and virus positive rates were analysed by the chi-square test. Associations between initial viral load and adverse outcomes of COVID-19 patients were identified using a multivariable logistic regression model. A two-sided *P *< 0.05 was considered to be statistically significant. All statistical analyses were conducted using SPSS version 19.0 and R version 3.6.1.

### Ethics approval

This study was approved by the Institutional Review Board of Jin Yin-Tan Hospital (KY-2020-02.01). Written consent was obtained from the guardians or legal representatives of patients.

### Role of the funding sources

The funder had no role in the study design, data collection, data analysis, data interpretation, or writing of the report. The corresponding author had full access to all data in the study and had final responsibility for the decision to submit the manuscript for publication.

## Results

### Virus positive rates in different kinds of samples

**Figure 1.**
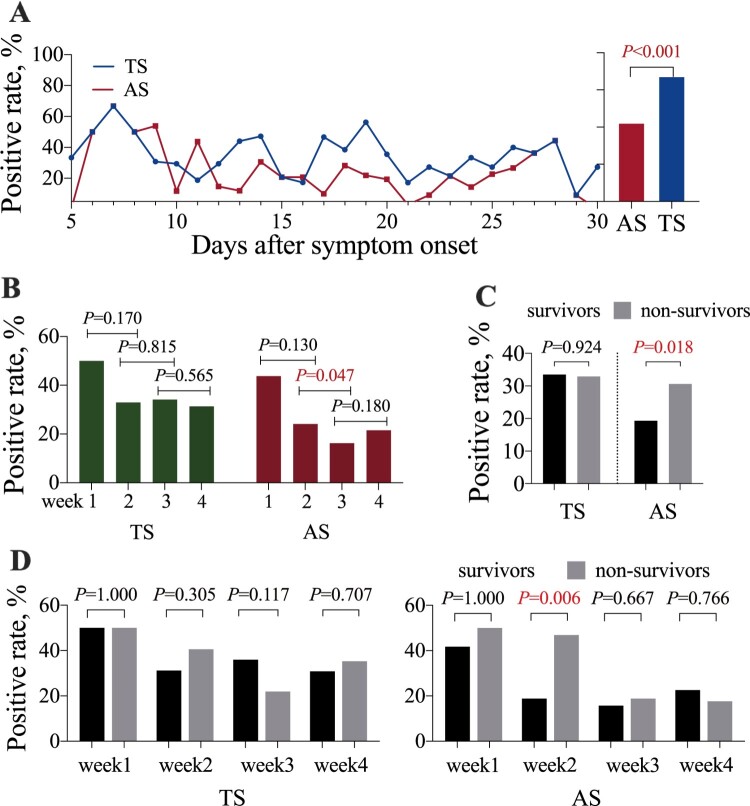
Virus positive rates in throat swab (TS) and anal swab (AS) samples. (A) The time course of positive rates in TS and AS samples on each day after symptom onset is shown (left part). The average viral RNA positive rates in TS and AS samples shown in the column were compared (right part). (B) The weekly positive rates in TS and AS samples after symptom onset. (C) The virus positive rates in TS and AS samples in survivors and non-survivors. (D) The weekly positive rates in survivors and non-survivors.

Viral RNA was detected in 314 samples (27.2%) among the 1156 samples tested, including 193 (33.4%) TS and 121 (20.9%) AS samples. The positive rate in TS samples was higher than that in AS samples (*P *< 0.001, chi-square test). The time to detectable viral RNA was 5 and 6 days post symptom onset (PSO) in TS and AS samples, respectively (Figure 1(A)). The positive rates in TS and AS samples reached a peak of 66.0% at day 7 PSO and then declined slowly (Figure 1(A)). The longest duration of viral RNA detection from symptom onset in TS was 39 days, and that in AS was 31 days. The positive rate in TS was not significantly different between the weeks of the study. The only noted difference was a decrease in the positive rate in AS in week 3 compared to week 2 PSO (*P* = 0.047) (Figure 1(B)). There was no association between death and prolonged detection of viral RNA in all types of specimens (≥20 days from illness onset). The mean positive rate in AS was higher in non-survivors than in survivors (*P* = 0.018) (Figure 1(C)), but a significant difference (AS, 46.9 *vs* 18.8, *P* = 0.006, chi-square test) was shown only in week 2 PSO (Figure 1(D)). The viral load in AS showed no correlation with symptoms of intestinal infections in our study (*P* = 0.255). Our data showed that the positive rate in AS was higher in non-survivors than in survivors.

### Time to positive detection in patients

SARS-CoV-2 RNA was detected in a total of 87 patients at the time of recruitment for this study. At least one kind of sample was positive for viral RNA in these patients, with 68 patients having a positive result from a TS and 44 having a positive result from an AS. Both samples showed viral RNA positivity in 25 patients.

**Figure 2.**
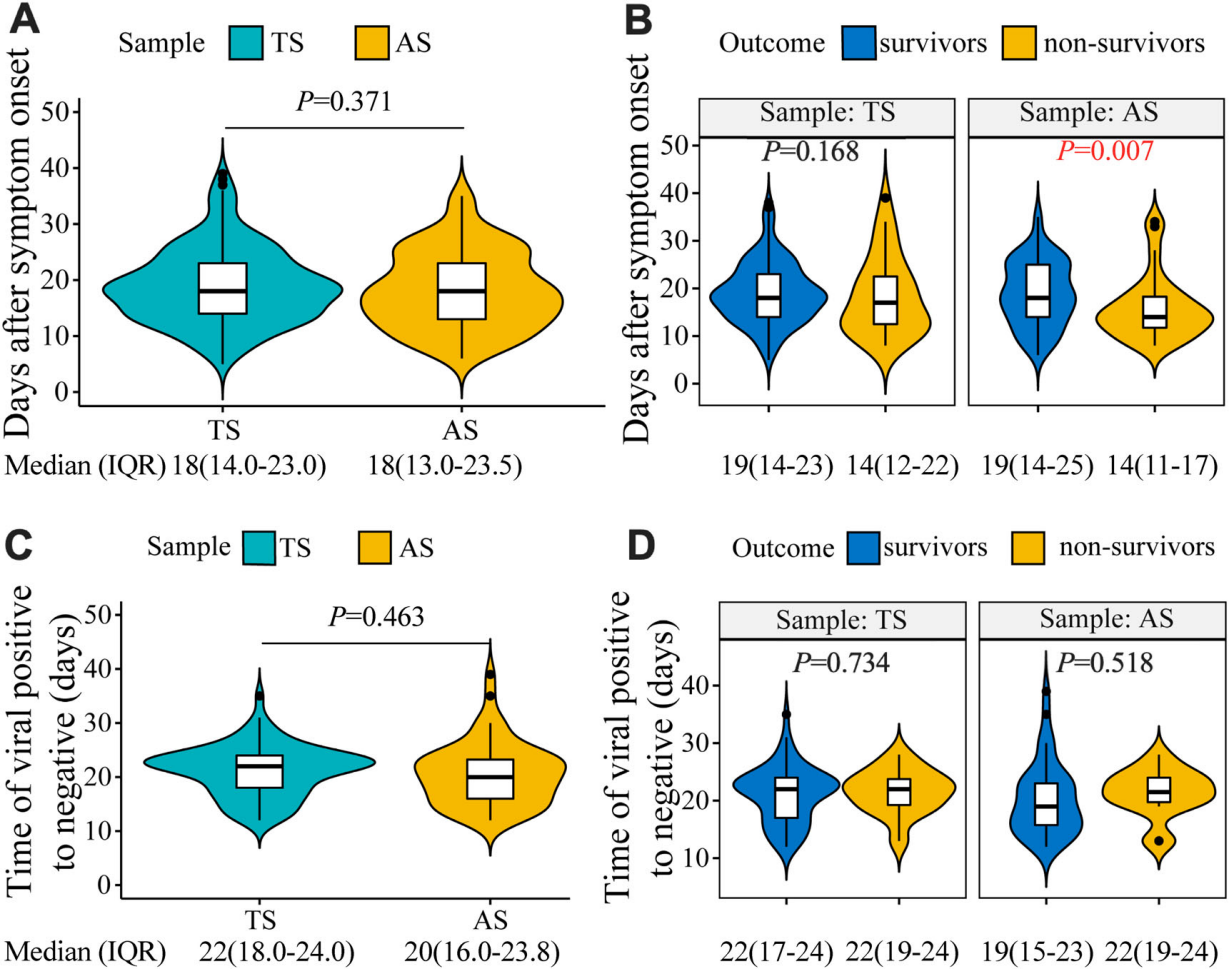
The time to SARS-CoV-2 positivity in throat swab (TS) and anal swab (AS) samples. (A) The time from symptom onset to viral RNA positivity in TS and AS samples. (B) The time from symptom onset to viral RNA positivity in TS and AS samples from survivors and non-survivors. (C) The time to viral RNA negativity after a positive result in TS and AS samples. (D) The time to viral RNA negativity after a positive result in TS and AS samples from survivors and non-survivors.

The median time to viral RNA detection was similar for TS [18.0 days PSO (IQR 14.0–23.0 days)] and AS [18.0 days PSO (IQR 13.0–23.5 days)] (Figure 2(A)). In non-survivors, the median time to viral RNA detection for AS (median number of days 14 *vs* 19 PSO, *P* = 0.007) but not TS (*P* = 0.168), was significantly earlier than that in survivors (Figure 2(B)). The time to viral RNA negativity after a positive result in TS and AS were similar in both survivors and non-survivors, with median times of 22.0 and 20.0 days PSO, respectively (Figure 2(C,D)). Our data showed that viral RNA in AS was detected earlier in non-survivors than in survivors.

### Kinetics of the viral load in patients

**Figure 3.**
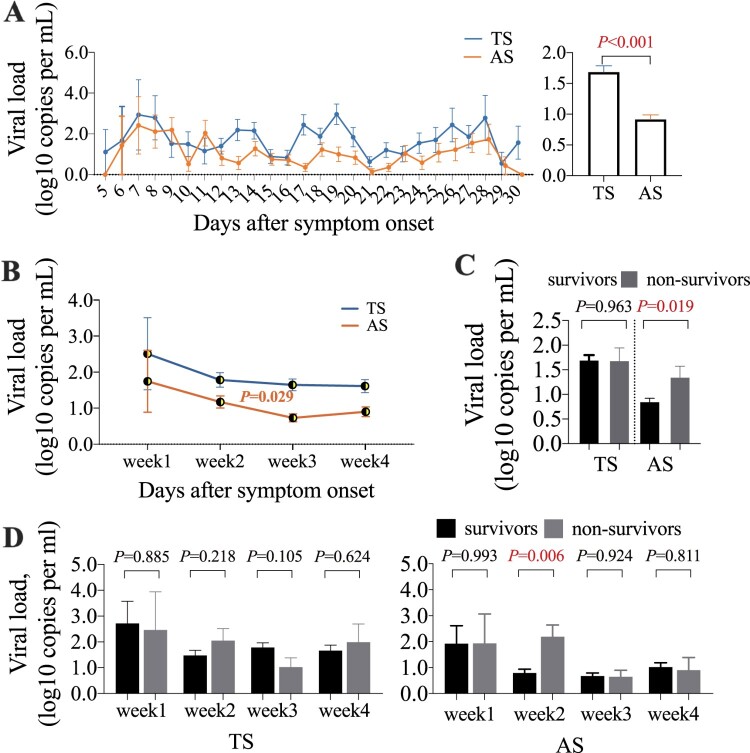
Viral load dynamics in throat swab (TS) and anal swab (AS) samples from patients with COVID-19. (A) The viral load in serial samples collected every 4–7 days. (B) The weekly mean viral load in the specimens post symptom onset. (C) The viral loads in specimens from survivors and non-survivors. (D) The weekly mean viral loads in specimens from patients with different outcomes post symptom onset.

The viral load in specimens ranged from 0 to 1.1×10^8^ copies/ml, with mean values of 1.0×10^6^ copies/ml in TS and 2.3×10^5^ copies/ml in AS. TS showed a higher viral load than AS (*P*<0.001) (Figure 3(A)). The TS viral load showed no significant difference between weeks PSO. The AS viral load in week 3 was significantly decreased compared to that in week 2 (*P* = 0.029) (Figure 3(B)).

The mean AS viral RNA load in non-survivors was approximately 5.6-fold higher than that in survivors (*P* = 0.019), particularly in week 2 PSO (*P* = 0.006) (Figure 3(C,D)). A high initial viral load in AS was associated with death (OR 1.368, 95% CI 1.076–1.741, *P* = 0.011), admission to the ICU (OR 1.237, 95% CI 1.001–1.528, *P* = 0.049) and need for invasive mechanical ventilation (OR 1.340, 95% CI 1.076–1.669, *P* = 0.009) in COVID-19 patients according to multivariable logistic regression model analysis after adjusting for age, severity based on the seven-category ordinal scale, use of corticosteroids, use of lopinavir-ritonavir and days from symptom onset to enrolment (Table 2). The AS (*P* = 0.402) and TS (*P* = 0.979) viral loads showed no significant difference between patients treated with or without lopinavir-ritonavir. The viral load reflects dynamic changes in viral replication and clearance by host immune activities. Our results showed that the patients who had adverse outcomes had higher AS viral loads than those patients who did not have adverse outcomes. These findings emphasize that enteric viral replication and transmission are important predictors of adverse outcomes.

**Table 2.** Association between initial viral load and adverse outcomes of COVID-19 patients.

Outcome	Initial viral load^†^ (log_10_ copies/ml)	Unadjusted OR (95% CI)	*P* value	Adjusted^††^ OR (95% CI)	*P* value
Death	TS	1.075 (0.936-1.235)	0.308	1.063 (0.891-1.268)	0.495
	AS	1.191 (1.002-1.415)	***0***.***047***	1.368 (1.076-1.741)	***0***.***011***
ICU	TS	0.960 (0.817-1.128)	0.622	0.938 (0.784-1.121)	0.481
	AS	1.204 (1.001-1.447)	***0***.***049***	1.237 (1.001-1.528)	***0***.***049***
IMV	TS	0.966 (0.815-1.146)	0.694	0.934 (0.773-1.128)	0.477
	AS	1.293 (1.071-1.562)	***0***.***008***	1.340 (1.076-1.669)	***0***.***009***

TS = Throat swab. AS = Anal swab. IMV = Invasive mechanical ventilation. ICU = Intensive care unit. OR = Odds ratio. † Initial viral loads in anal swab and throat swab samples were analysed. †† Results were adjusted based on age, severity on the seven-category ordinal scale, use of corticosteroids, use of lopinavir-ritonavir and days from symptom onset to enrolment.

## Discussion

In this study, we analysed the viral RNA positive rate and viral loads in consecutively collected paired TS and AS samples from hospitalized severe COVID-19 patients. We found that viral RNA could be detected in TS and AS samples, but the rates of positivity were different (TS 33.4%, AS 20.9%). The mean viral loads were also different between groups (TS 1.0×10^6^ copies/ml, AS 2.3×10^5^ copies/ml). The time from symptom onset to positive viral RNA detection in AS samples was significantly lower in non-survivors than in survivors (median number of days of 14 *vs* 19). The virus positive rate and the viral load in AS in week 2 after symptom onset were significantly higher in non-survivors than in survivors.

Several groups have reported the detection rate of SARS-CoV-2 in different samples from COVID-19 patients with different disease severities. However, the data vary greatly between studies [11,12,15]. Other studies reported that the average viral RNA positive rates in TS and faecal samples from COVID-19 patients were 18.2%–62.5% and 17.0%–26.7%, respectively [12,16]. These disparate values may be attributed to the inconsistent disease severity, sampling time, sample number and type used to evaluate the viral positive detection rates across studies. The TS viral load between weeks showed no significant difference PSO in our study, which was consistent with Zheng et al.’s study [7]. But Wölfel et al.’s considered there were significant differences of TS viral load between weeks PSO [14]. The difference between our findings and those of previous reports may related to the enrolled cases with different disease severities.

The viral RNA detections in enteric samples were similar to that in SARS patients, in which the virus was isolated from stool samples, and a high viral RNA prevalence was found in the stool samples [17,18]. Human organoid culture experiments have shown that replication of SARS-CoV-2 in the gut is higher than that in the lungs [19,20]. The expression of N protein was visualized in the cytoplasm of gastric, duodenal, and rectal glandular epithelial cells, which further confirmed the regional replication of SARS-CoV-2 [21]. The presence of viral RNA in different anatomical sites indicates the location of replication and/or the transmission route. It is well known that the respiratory tract is the initial replication site of SARS-CoV-2. The detection of viral RNA in anal samples might be the result from transmission of virus from the respiratory tract to the intestinal tract by swallowing, the replication of virus within extrapulmonary organs, or the increased intestinal permeability during disease progression. However, we found no correlation of the viral load in AS with intestinal infection symptoms in our study, though diarrohea and vomiting were reported in the COVID-19 patients [15]. To obtain proof of active virus replication in the absence of histopathology, we also analysed viral subgenomic mRNAs in clinical samples [14]. It showed that subgenomic mRNAs were detectable in both viral RNA positive TS (35.6%) and AS (13.9%) samples (Supplementary Figure 1). Our viral shedding data also indicate the important role of the gut during disease progression. Collectively, these findings emphasize that enteric viral replication and transmission in individuals are important predictors of disease severity. Enteric samples should be routinely collected for virally testing for COVID-19 diagnosis, as they are for SARS diagnosis.

The viral load reflects the dynamic interplay between viral replication and virus clearance by host immune activities [18]. The examination of viral load in SARS and Middle East Respiratory Syndrome (MERS) patients has been used to predict disease progression [10,22]. In SARS, a high viral load in respiratory, stool and blood samples was related to death [10]. In MERS, the viral loads in the severe group were higher than those in the mild group, while the viral shedding time and intensity were closely related to SARS [22,23]. The viral load in the respiratory tract was reported positively linked to lung disease severity in COVID-19 patients, indicating that it is a predictor of disease severity [24]. In our data, the TS viral load was higher than the AS viral load. However, we found no correlations of TS viral load with death in our study. Such finding was consistent with Fajnzylber et al’s report, in which they recruited severe COVID-19 patients [25]. The disparities might be related to the disease severity of recruited patients, sampling time and sample number. Only the AS viral load was significantly higher in non-survivors than in survivors, and the difference became significant at week 2 PSO, which may indicate that the second week during disease progression is a critical point for determining COVID-19 outcome. The presence or absence of an extrapulmonary infection at week 2 indicates whether a patient's immune system has been effective in preventing the spread of the virus, thereby determining the patient's chance of survival.

The viral RNA detections and viral load in consecutively collected paired samples from patients showed that TS was positive in high viral RNA concentrations, followed by AS from survivors and non-survivors. The respiratory tract being the primary replication site of SARS-CoV-2 was supported by the high TS viral load. Viral RNA could be detected earlier in TS than AS, and TS had higher viral loads than AS in both survivors and non-survivors. The expression of angiotensin-converting enzyme 2 (ACE2), the receptor for SARS-CoV-2, is much higher in the small intestine than in the lungs [26–28]. It is hypothesized that in some patients, the virus travels to the intestine after the initial respiratory system infection and actively replicates [29]; viral RNA “spillover” into the blood would thus predict adverse outcomes. Monitoring enteric and blood samples would be a specific way to monitor disease progression.

There are some limitations of our study. One is that there was a lack of samples from the first 5 days, and as such, we could not provide a detailed characterization of viral load kinetics in the early stage. The second is that although the AS and TS were collected at the same time every 4–7 days until the patients were discharged or died, we did not continue to monitor the patients after discharge. However, it would be not affected the conclusions in this study. The third is that we analysed the dynamics of viral RNA positivity and viral load with samples taken from patients who received antivirals, antibiotics, corticosteroids and other treatments, which could have affected the patterns. However, our findings can help identify those patients with severe COVID-19 who are likely to experience disease progression.

In conclusion, based on the analysis of a relative large amount of samples collected from severe COVID-19 patients, we found that a high viral RNA positivity rate in AS, a high viral load in AS, and early positive detection in AS can predispose COVID-19 patients to adverse outcomes. Early administration of effective antiviral drugs is critical for treating COVID-19. The presence of viral replication in extrapulmonary sites predisposes to adverse outcomes and should thus be monitored carefully.

## Supplementary Material

FigS1.eps
